# The impact of the COVID-19, social distancing, and movement restrictions on crime in NSW, Australia

**DOI:** 10.1186/s40163-021-00160-x

**Published:** 2021-10-24

**Authors:** Joanna J. J. Wang, Thomas Fung, Donald Weatherburn

**Affiliations:** 1 School of Mathematical Physical Sciences, University of Technology, Sydney, Australia; 2 Department of Mathematics and Statistics, Macquarie University, Sydney, NSW Australia; 3 National Drug and Alcohol Research Centre, University of New South Wales, Sydney, Australia

**Keywords:** COVID-19, Lockdown, Assault, Theft, Robbery, Criminal opportunity, Domestic violence

## Abstract

The spread of COVID-19 has prompted Governments around the world to impose draconian restrictions on business activity, public transport, and public freedom of movement. The effect of these restrictions appears to vary from country to country and, in some cases, from one area to another within a country. This paper examines the impact of the COVID-19 restrictions imposed in New South Wales (NSW) by the State Government. We examine week-to-week changes in 13 categories of crime (and four aggregated categories) from 2 January 2017 to 28 June 2020. Rather than using the pre-intervention data to make a forecast and then comparing that with what is actually observed, we use a Box–Jenkins (ARIMA) approach to model the entire time series. Our results are broadly in accord with those of other studies, but we find no effect of the lockdown (upward or downward) on domestic assault.

**Supplementary Information:**

The online version contains supplementary material available at 10.1186/s40163-021-00160-x.

## Introduction

Our ability to disentangle cause and effect in crime is often hampered by the fact that many of the factors which affect crime (e.g., arrest rates, police numbers, penalty severity) are also affected by it. Every now and then, however, an event occurs that provides unique insight into the importance of some process as a generator of crime (e.g., Drago et al., 2007; Klick & Tabarrok, 2005). The emergence of COVID-19 and the Governmental response to COVID-19 is one such event. The restrictions placed by Governments on business activity and social interaction in response to COVID-19 (hereafter referred to as the COVID-19 restrictions) were not designed as a response to crime but did have a dramatic effect on the structure of opportunities, incentives, and triggers for involvement in crime. The opportunities and incentives for committing many types of crime (e.g., burglary, motor vehicle theft) were sharply reduced. At the same time, the rise in unemployment and financial stress resulting from these restrictions greatly increased the incentives for involvement in crime.

The net effect of these two forces is of considerable theoretical and practical significance. Traditional theories of crime suggest the explanation for variation in crime rates lies in terms of changes in the supply of motivated offenders. Rational choice and criminal opportunity theories, by contrast, take the supply of motivated offenders as a ‘given’, arguing that the primary driver of crime is the supply of opportunities for involvement in crime. The evidence accumulated since the formulation of these theories suggests that crime is a product of factors that affect both the supply of motivated offenders and the supply of criminal opportunities and incentives. This fact, however, takes us no closer to knowing which set of factors will dominate in any particular context. Deprived of opportunities to commit burglary, offenders might simply reduce their criminal activity or switch to other kinds of income-generating crime, such as fraud. Faced with unemployment, those who might otherwise rarely offend may deepen their involvement in income-generating crime. Theoretical predictions concerning violent crime are just as uncertain. Forcing families to spend longer periods together at home might result in an increase in domestic violence. Closing licensed premises and thereby reducing alcohol consumption may have the opposite effect.

The aim of this article is to report the results of a study into the effect of the COVID-19 restrictions on crime in New South Wales (NSW), Australia. The NSW experience is of interest for three reasons. Firstly, given the global nature of COVID-19, it is inherently of interest to know whether its effects on crime in countries that have very different social security systems and crime problems. Secondly, in contrast to some countries (Wang & Pagán, 2021), in the early stages of the COVID-19 epidemic, Australians on both sides of politics appeared willing to accept quite severe restrictions on business activity (including business closure), interstate travel and freedom of movement (including a requirement to stay at home) (Haseltine, 2021). Thirdly, as the results from studies of the effect of COVID-19 restrictions flow in from different countries (and jurisdictions), the precise effect of movement restrictions on crime may become clearer.

The remainder of this article is structured as follows. In the next section we summarize past research into the effect of COVID-19 (and associated behaviour) on crime. We then provide background information on the Australian and NSW Government responses to COVID-19 and describe the impact of the COVID-19 restrictions on social movement and business activity in NSW. The following two sections describe our approach to modelling the effect of COVID-19 restrictions on crime and present our findings. The final section summarizes the findings and puts forward recommendations for future research.

## Related literature

### U.S. research

Because initial studies of the effect of COVID-19 related restrictions had short follow-up periods, their results are somewhat conflicting. Ashby (2020), for example, employed seasonal ARIMA models to examine changes in six crime types (serious assaults in public places, serious assaults in residences, residential burglaries, non-residential burglaries, theft of vehicles and theft from vehicles) in 16 large U.S. cities. The ARIMA models were used to generate forecasts for each crime type, which were then compared to observed crime trends from the 20th of January 2020 (the date of the first confirmed COVID case) to the 10th of May. He found no significant changes in the frequency of assaults in public, or in residences, nor any change in non-residential burglary up to the 20th of May 2020. Significant reductions in motor vehicle theft and theft from motor vehicles were observed, but only in some of the 16 cities.

Mohler et. al. (2020) examined daily counts of calls for service from 2 January 2020 to 18 April 2020 in Los Angeles and from 2 January 2020 to 21 April 2020 in Indianapolis, before ‘shelter-in-place’ (stay at home) orders were issued in these cities. They found a significant reduction in burglary and robbery in Los Angeles but no significant reduction in either offence in Indianapolis. Neither city experienced an increase in calls related to assault/battery, but both experienced an increase in calls for assistance related to domestic assault. Calls related to vehicle theft rose in Los Angeles but remained stable in Indianapolis. Vandalism calls also moved in opposite directions in Los Angeles (down) and Indianapolis (up). Mohler et. al. (2020, p. 1) conclude that while ‘social distancing has had a statistically significant effect on a few crime types [the] overall effect is notably less than might be expected given the scale of disruption to social and economic life.’

Abrams (2021) examined crime changes in response to the issuing of stay-at-home (SAH) orders in 25 of the largest U.S. cities using a difference-in-difference strategy where the SAH counterfactual in each city was the trend in crime in that city in the years prior to 2020. In the period between the issue of stay-at-home orders and the end of May he found a substantial (23.3%) fall in total recorded crime, most of which was driven by falls in residential burglary (down 23.5%), other theft offences (down 33%), robbery (down 20.2%) and simple assault (down 33.3%). The fall in residential burglary was offset by a rise in non-residential burglary (up 37.8 per cent). No change was observed in the incidence of vehicle theft, however there were falls in domestic assault (down 17.3%) and sexual assault (down 38.6%).

The Mohler et. al. (2020) and Abrams (2021) studies revealed significant differences between U.S. cities in the effects of COVID-19 and associated restrictions. Campedelli et. al. (2020) found marked differences across communities even within a city. They examined changes in burglary, assault, narcotics offences, and robbery between 2019 and 2020 across 77 communities in Chicago. The vast majority of communities did not experience any significant change in crime, while among those that did experience a change, several experienced an increase. The response to the COVID-19 restrictions also varied across crime types, with 12 per cent of communities experiencing a fall in robbery, 13 per cent of the communities experiencing a drop in burglary, 23 per cent experiencing a fall in assault and 46 per cent experiencing a fall in narcotics-related offences.

A number of commentators have expressed concern about the potential impact of COVID-19 stay-at-home restrictions in the incidence of domestic assault. Piquero et. al. (2020) examined this issue using data from Dallas, Texas. They found some evidence of a short-term spike in family violence in the 2 weeks after the stay-at-home order was issued but a decrease thereafter. The short post-intervention time series, however, made it difficult to determine whether the increase in calls for service after the stay-at-home order was a genuine effect or the continuation of a pre-existing trend. Bullinger et. al. (2020) followed Abrams in conducting a difference in difference analysis of domestic violence calls for service, using the period leading up to the imposition of the stay-at-home order in Chicago as the control group. They found a rise in calls for service related to domestic assault but, somewhat confusingly, a decrease in reports and arrests for domestic assault. More recently, Piquero et. al. (2021) conducted a systematic review and meta-analysis of studies that have examined the impact of COVID-19 on domestic assault and found that most estimates suggested a significant positive (upward) effect of the lockdown on this offence.

### Research in other countries

Although most of the research on the impact of COVID-19 restrictions has focussed on the U.S., studies have also been conducted in Sweden, the United Kingdom, Canada, and Australia. Sweden is of interest because that country initially chose not to implement the dramatic restrictions on social movement and business activity adopted by other countries. Gerell et. al. (2020) examined weekly counts of assault (‘indoors’ and ‘outdoors’), personal robbery, residential burglary, commercial burglary, narcotics crime, pickpocketing and vandalism before and after the first of the COVID-19 recommendations for social distancing was issued by the Swedish government. They observed modest falls in (indoor and outdoor) assaults, residential and commercial burglary, and a large fall in pickpocketing but no change in personal robbery or narcotic related crime.

Halford et. al. (2020) studied the effect of the UK COVID-19 restrictions on total recorded crime and the frequency of a number of specific offences, including shoplifting, other theft, domestic abuse, theft from a vehicle assault, burglary (non-dwelling and dwelling) and motor vehicle theft. As in several other studies, they used data on trends in recorded crime prior to the COVID-19 restrictions to develop forecasts of the expected trend in crime after the movement restrictions were introduced. They then compared the observed trend in crime to these forecasts on the assumption that any discrepancy between predicted and observed trends in crime represented the effect of the COVID-19 and associated restrictions. They observed significant and substantial reductions in all categories of crime except motor vehicle theft.

Langton et. al. (2021) have recently examined crime trends in England and Wales 6 months after the nationwide lockdown in response to COVID. They compared observed with expected crime trends for fourteen different offence categories between March and August, 2020. The expected trends were generated using a set of ARIMA-based short-term forecasts estimated using recorded crime data from March 2015 to August 2020. They found that most crime types experienced sharp, short-term declines during the first full month of lockdown, followed by a gradual resurgence as restrictions were relaxed. By the end of March, anti-social behaviour and drug crimes, however, had both increased. Anti-social behaviour was described by the authors has having ‘skyrocketed’ (p. 8).

Hodgkinson and Andresen (2020) employed interrupted time series analysis to examine crime trends in Vancouver, Canada, in the 12 weeks following the 18th of March 2020 declaration of a public health emergency by the provincial government in British Columbia and the subsequent introduction of measures designed to limit the spread of COVID-19. As with the study by Halford et. al. (2020), they constructed a model using data from a pre-COVID-19 period (beginning on the 29th of May 2017) to generate crime forecasts for the post-COVID-19 period and then compared the observed with the forecast trends. They found lower than forecast rates of total crime, theft from vehicle and general theft but no change in violence or ‘mischief.’ Auto theft was found to be stable when the forecast model suggested it should have been increasing, but commercial burglary first increased and then decreased.

Several studies of the effect of COVID-19 on crime in the state of Queensland, Australia have been published. Andresen and Hodgkinson (2020) employed structural break analysis to examine the impact of the COVID-19 pandemic on various categories of crime in rural, regional and urban settings in Queensland over the period May 2018 to 02 July, 2020. Like Langton et. al. (2021), they found that recorded crime decreased during the initial lockdown, but increased once social restrictions were relaxed. In some parts of Queensland, however, rates of violent offending increased. McCarthy et. al. (2021) examined changes in youth offending in Queensland, over the period January 2018 and 30 June 2020. Their panel regression indicated significant declines in youth property offending, offences against the person and public order offences, but no significant changes in illicit drug offences. Other research using Queensland data, however, indicates that overall drug offences increased (Langfield et al., 2021).

Payne et. al. (2021) examined the impact of the COVID-19 restrictions on police recorded numbers of common assaults, serious assaults, and sexual offences in Queensland, comparing ARIMA based forecasts of trends in these offences with the actual numbers observed after March 2020, the month in which Queensland first introduced social distancing requirements. That study found significantly lower rates of serious assault and sexual offences than forecast. The frequency of common assault also fell below the level forecast, although the decline was not statistically significant. Payne et. al. (2021) carried out a similar analysis of changes in property damage, shop stealing, burglary, motor vehicle theft and credit card fraud in Queensland over the same time period as used in the Payne et. al. (2020) study. They found significant reductions in shop theft, other theft, and credit card fraud but no significant change in property damage, burglary, and motor vehicle theft. Kim and Leung (2020) obtained somewhat different results in their examination of changes in crime in the 6 week period between the 15th of March 2020 and the 26th of April 2020. That study reported lower than forecast rates of non-domestic assault, sexual offences, robberies, residential break-ins, non-residential break-ins, vehicle theft, stealing from a vehicle and retail theft.

The final and perhaps the most important study conducted to date on the COVID-19 crime relationship is that published by Nivette et. al. (2021). They collected data on daily counts of crime across 27 cities in 23 countries in the Americas, Europe, the Middle East and Asia and conducted an interrupted time series analysis to assess the impact of stay at home restrictions on different types of crime in each city. Reflecting what we have already seen in this review, they found substantial variations in the effect of these restrictions across cities and types of crime. They then ranked cities according to the stringency of their stay at home restrictions on a scale of 0 (no measures) to 3 (do not leave the house with minimum exceptions). Using mixed effect meta-regression methods they were able to show that larger crime suppression effects occurred in cities with more stringent stay at home restrictions.

## Background to the current study

The aim of the current study is to contribute to the growing body of research on the effects of COVID-19 and associated restrictions on trends in crime. Our focus is on the effect of COVID-19 and associated restrictions on crime in NSW. In this section, we describe the response of the NSW Government to the pandemic and its effect on social activity.

The World Health Organisation declared a global pandemic on the 11th of March 2020. On the 15th of March 2020, NSW residents were advised to work from home if possible, avoid crowds and gatherings, reduce their use of public transport, and keep 1.5 m away from other people. Three days later, non-essential indoor gatherings were formally limited to 100 people, while outdoor gatherings (including all sporting and entertainment fixtures) were limited to 500 people. Organizers of indoor gatherings were also required to set aside four square metres per person. Five days later, all non-essential businesses were closed, including licensed venues, gyms, cinemas, restaurants, cafes, and places of worship. The following day, all schools were closed, and students moved to online teaching. Finally, on 31 March, criminal offences were enacted, making it an offence for a person to leave their place of residence without a reasonable excuse. The same legislation limited outdoor gatherings to two people.

**Fig. 1:**
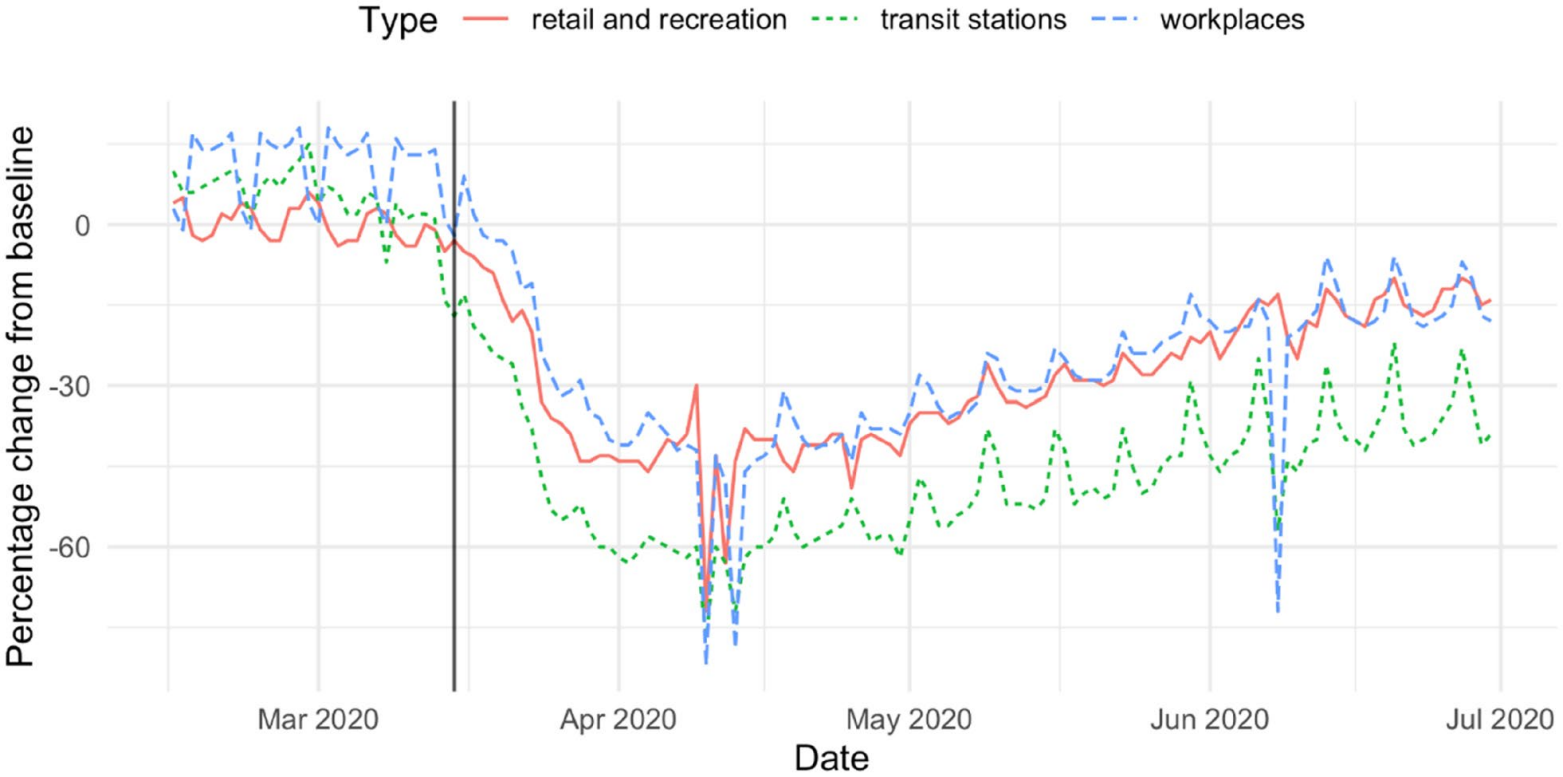
Percentage change in three Google mobility measurements relative to a baseline that is the median day-value from a 5-week period in January

Figure 1 shows the percentage change in three Google mobility measurements relative to a baseline that is the median day-value from a 5-week period in January (Google 2021).

There is a rapid decline in all three indices, followed by a slow return toward ‘normal’. There is some evidence the decline began prior to the announcement of the pandemic, but most of the fall in mobility happens between the 15th of March 2020, when NSW residents were advised to work from home if possible, avoid crowds/gatherings and reduce their use of public transport and 31 March, when it became an offence for a person to leave their place of residence without a reasonable excuse and outdoor gatherings to two people. By the beginning of April, transit station mobility had declined by around 60 per cent, while workplace, retail, and recreation mobility had declined by around 40 per cent. The COVID-19 restrictions (and the announcement of a pandemic) clearly had a substantial suppression effect on social activity in NSW.

To examine the effect of these changes (hereafter referred to as “lockdown” or “intervention”) on crime in NSW, we examine trends in 13 specific offences: break and enter (dwelling), break and enter (non-dwelling), motor vehicle theft, stealing from a dwelling, stealing from the person, sexual assault, indecent assault/act of indecency, robbery, domestic assault, non-domestic assault, and fraud (most of which is identity theft). We do not examine drug offences and other offences discovered by (rather than reported to) police because large numbers of police during the study period were shifted from routine policing to enforcement of COVID-19 restrictions. This would have limited their capacity to engage in the routine surveillance and patrol activity (e.g., stop and search) that would normally lead to the discovery of offences such as drug possession and fare evasion. Our study differs in another important respect from earlier research. As already noted, most studies have used a post-COVID-19 crime forecast as the counterfactual against which to gauge the effect of the COVID-19 restrictions on crime. The main problem with this approach is that the crime predictions will become less accurate (i.e., have larger standard errors) as time increases. We use a Box–Jenkins (ARIMA) modelling approach, but rather than using just the pre-intervention data to make a forecast (“what would have been expected given the pre-intervention trend”) and then comparing that with what is actually observed, we model the entire time series (both pre- and post-intervention) and incorporate level and trend change terms in the model. This gives us a clearer picture of the nature of any crime change following the introduction of the COVID-19 restrictions. In the next section of this article, we describe the methods employed in more detail.

## Method

As previously noted, we use weekly counts of crime incidents reported to or detected by the NSW Police Force from the week beginning 2 January 2017 to the week ending 28 June 2020. We chose this date as the end of the study period as the initial COVID-19 lockdown restrictions which commenced in March 2020 was in force for the maximum 90-day period permitted under NSW law until the end of June 2020. These data were extracted from the NSW Police Force’s Computerised Operational Policing System and provided by the Bureau of Crime Statistics and Research.

In the analyses below, we consider the following crime categories:

- Domestic violence (DV) related assault
- Non-domestic violence related assault
- Sexual assault; indecent assault/act of indecency. Collectively referred to as ‘aggregated sexual offence’
- Robbery with a weapon not a firearm; robbery without a weapon. Collectively referred to as ‘aggregated robbery’
- Break and enter dwelling; break and enter non-dwelling; motor vehicle theft; steal from motor vehicle; steal from dwelling; steal from person. Collectively referred to as ‘aggregated theft’
- Fraud

Note that our data on each individual crime type are nested within the aggregated groups. In fact, multiple crime time series are often hierarchically organised and can be aggregated at several different levels in groups based on crime type or geographical location. Disaggregating crime into its components is often recommended. This helps to identify any more subtle changes than total crime, but authorities and decision-makers are often only interested in results at the higher levels of aggregation, i.e. the big picture. It is natural to want the estimates to add up in the same way as the data would as you go up the hierarchical structure. This rules out fitting all disaggregate and aggregate series independently as there is no way to guarantee the forecasts will consistently add up between aggregation levels. One common solution is to forecast only the most disaggregated series and summing the results, but this often leads to poor forecasting performance at the higher levels of aggregation in practice as the most disaggregated series often have a high degree of volatility while the most aggregated time series is usually smooth and less noisy. The method implemented in this paper (see Wickramasuriya et al., 2019 for instance) will provide the best of both worlds. Not only it will provide forecasts on all disaggregated series, it will also provide forecasts that add up appropriately across the hierarchy, and are also unbiased and have minimum variance amongst all combination forecasts. The optimality is achieved using all the information available such as relationships between series within a hierarchical structure.

## Analytical model

Interrupted time series (ITS) analysis is a valuable quasi-experimental study design for evaluating the effectiveness of population-level interventions that have been implemented at a clearly defined point in time. ITS methods are increasingly being used in evaluating policy changes and programs in criminology research (Piehl et al., 2003; Vujić et al., 2016) and we use this approach in the current study. In standard ITS analyses, a segmented regression model capturing underlying pre-intervention trend and level and slope change following the intervention is used. However, a number of distinct features of time series data must be addressed when using ITS. This includes autocorrelation and seasonality. To accounts for these features, we assume an ARIMA structure holds for the series of interest, both before and after the intervention. A non-seasonal ARIMA model $ARIMA\left( {p,d,q} \right)$ can be written as

1 $$ \left( {1 - \phi_{1} B - \cdots - \phi_{p} B^{p} } \right)\left( {1 - B} \right)^{d} y_{t} = c_{t} + \left( {1 + \theta_{1} B + \ldots + \theta_{q} B^{q} } \right)\varepsilon_{t} , $$

where in our case $y_{t}$ corresponds to each weekly crime incident type; $p$ is the order of the autoregressive component; $d$ is the degree of differencing involved; $q$ is the order of the moving average component, $B$ is the backshift operator and $\varepsilon_{t}$ is the normally distributed forecasting error that shows no autocorrelation. The constant component $c_{t}$ consists of:

2 $$ c_{t} = \beta_{0} + \beta_{1} Time_{t} + \beta_{2} Lockdown + \beta_{3} Time_{t} \times Lockdown, $$

where $Time_{t}$ represents weekly intervals and was treated as a continuous variable cantered on 15 March, $Lockdown$ is an indicator variable that takes the value of 0 prior to 15 March 2020, and 1 after the lockdown order went into effect. In the above model, $\beta_{0}$ estimates the level of each crime incident type prior to the lockdown; $\beta_{1}$ estimates the pre-lockdown time trend; $\beta_{2}$ estimates the change in the intercept immediately after the lockdown and $\beta_{3}$ estimates the change in trend. Since time is centred on the date that the lockdown order came into effect, $\beta_{2}$ also represents the invention effect.

We include additional seasonal terms in the ARIMA model above and characterize it as an $ARIMA\left( {p,d,q} \right)\left( {P,D,Q} \right)_{m}$. The uppercase notation is used to represent the orders of the seasonal components of the model and $m$ defines the seasonal period, which equals to 52 for our weekly data. For example, an $ARIMA\left( {1,1,1} \right)\left( {1,1,1} \right)_{52}$ model (without a constant) for weekly data can be written as

$$ \left( {1 - \phi_{1} B} \right)\left( {1 - \Phi_{1} B^{52} } \right)\left( {1 - B} \right)\left( {1 - B^{52} } \right)y_{t} = \left( {1 + \theta_{1} B} \right)\left( {1 + \Theta_{1} B^{52} } \right)\varepsilon_{t} . $$

The orders of the seasonal ARIMA interrupted time series model were chosen based on the corrected Akaike Information Criterion (AIC_c_). The independence of the model residuals was checked using the Box–Ljung test (Ljung & Box, 1978). All model estimation is undertaken using R with the fable package (O’Hara-Wild et al., 2020).

## Results

**Fig. 2:**
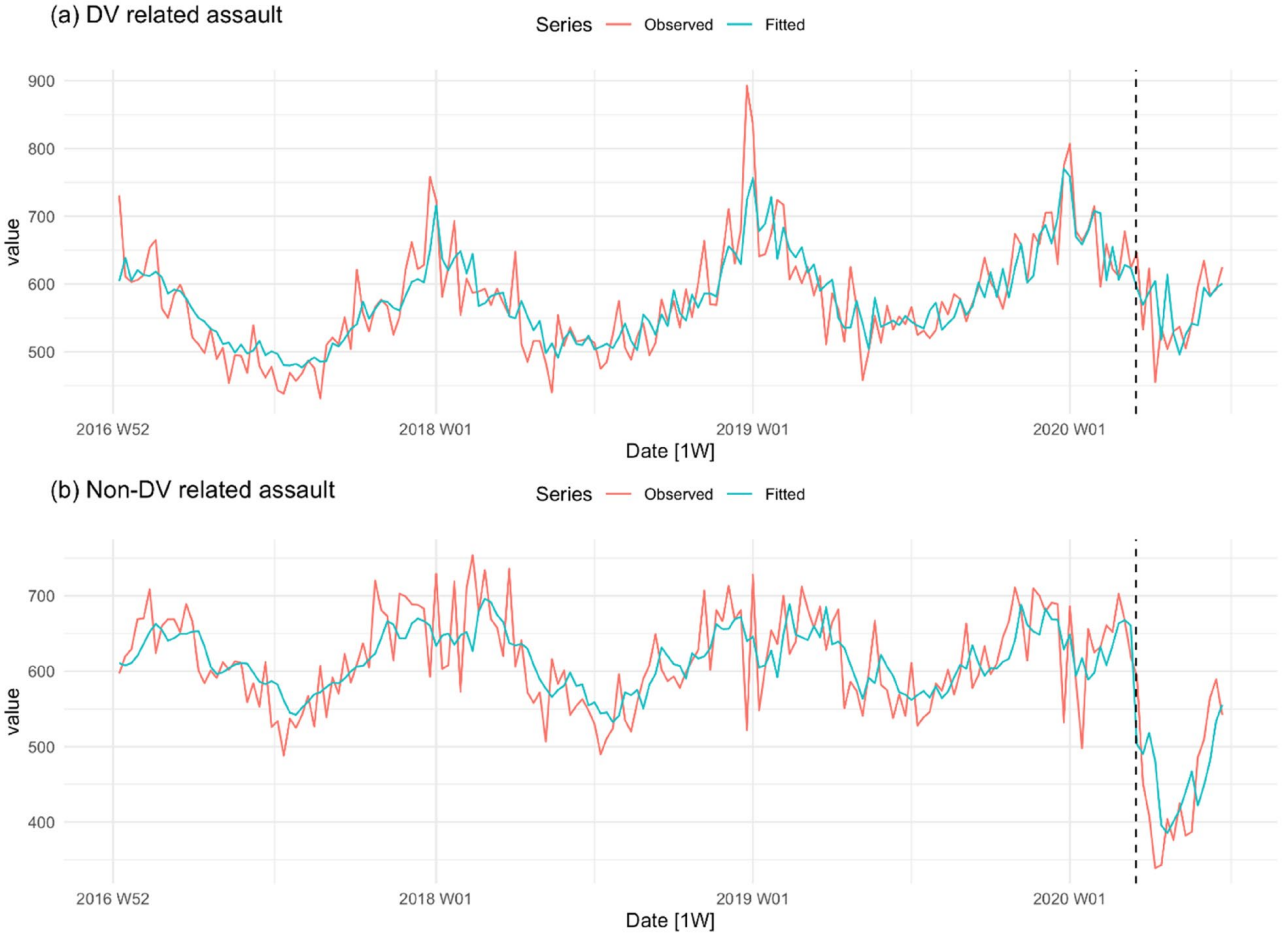
DV and non-DV related assaults per week in NSW

We begin with a visual examination of the trends in crime in the pre- and post-COVID-19 lockdown periods. The minimum and maximum values of each time series are given in Table 1. Figure 2 shows the observed and fitted trends in domestic violence (DV) and non-domestic violence (non-DV) assault. There are strong seasonal effects in both series in the pre-COVID-19 period, with the incidence of both types of assault rising rapidly in summer and falling rapidly in winter (June, July and August in Australia). Across the whole of the pre-lockdown period, however, the mean level of the series appears stable. In the case of DV assault, the decline in the post-lockdown period (approaching winter) seems consistent with similar downward trends at the same time in earlier years. The abrupt post-lockdown drop in the level of non-DV assault, however, appears much sharper than in earlier years.

**Table 1:** Minimum and maximum values for each series

Crime type/group	Minimum	Maximum
Domestic assault	431	893
Non DV assault	339	754
Indecent assault/act of indecency	83	220
Sexual assault	68	176
Aggregated sexual/indecent assault	156	366
Robbery with a weapon not a firearm	7	34
Robbery without a weapon	14	43
Aggregated robbery	23	68
Break and enter (dwelling)	259	656
Break and enter (non-dwelling)	76	256
Motor vehicle theft	156	320
Steal from motor vehicle	353	893
Steal from dwelling	257	471
Steal from person	15	115
Aggregated theft	1123	2494
Fraud	630	1525
Total crime	2989	5235

**Fig. 3:**
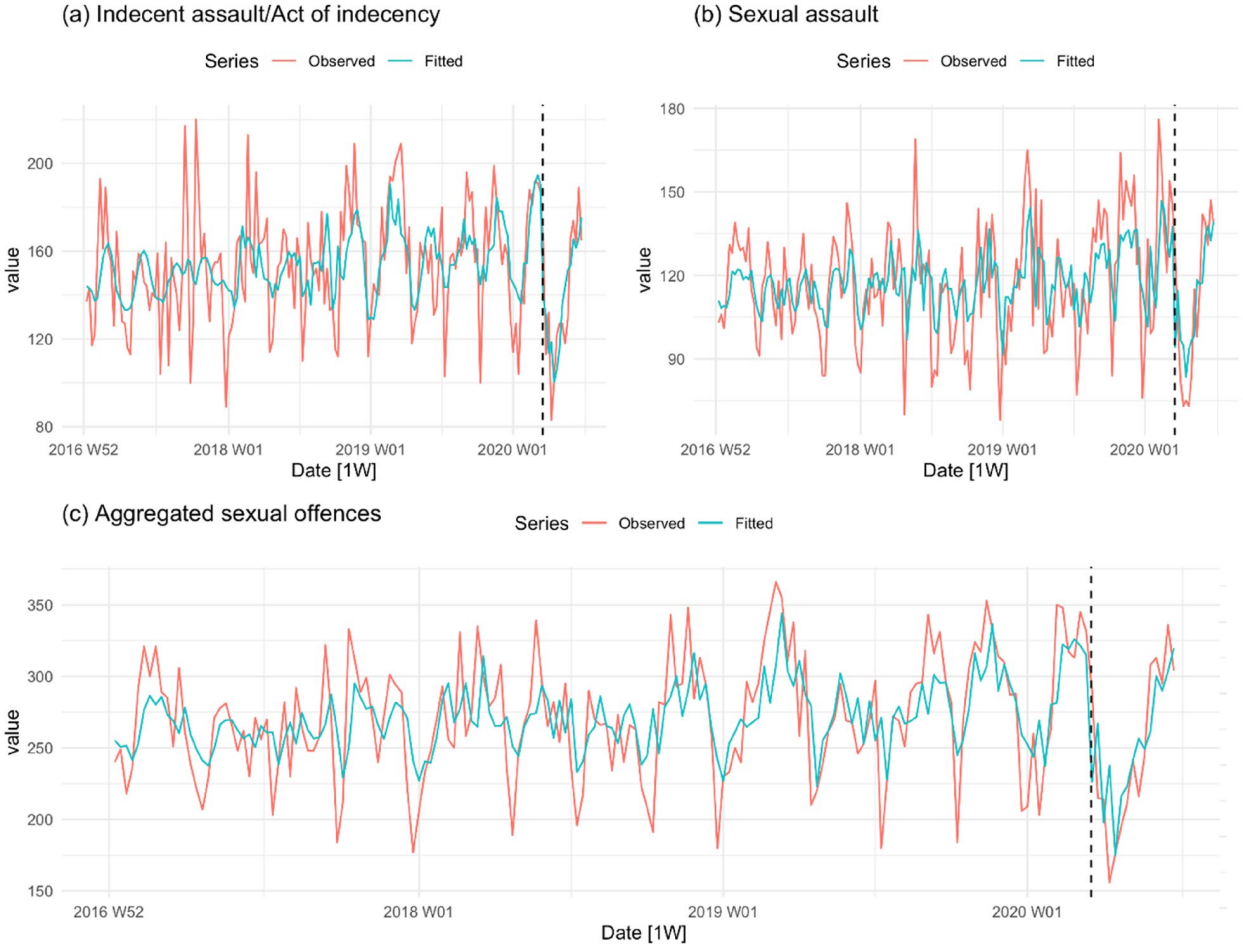
Indecent assault, sexual assault, and aggregated sexual assault per week in NSW

Figure 3 shows the corresponding trends for indecent assault, sexual assault, and aggregated sexual assault. As with non-DV and DV related assaults, there are strong seasonal effects in the pre-COVID-19 period. In this case, however, the mean level of all three series appears to be slowly increasing. Close inspection of the trend for indecent assault/act of indecency shows an abrupt decline in level with the onset of the lockdown and an equally sharp rebound. Although there are similar dips in reported offences at the same time of the year in earlier years, the change in level after the lockdown appears larger than normal. The observed and fitted values for sexual assault are similar to those for indecent assault/act of indecency, however the drop in reported incidents in the post-COVID-19 period is less pronounced. The aggregated series is suggestive of COVID-19 effect, with the observed sex offence series falling from a peak of 325–350 incidents a week immediately prior to the lockdown to a low point of a little over 150 incidents a week immediately after the lockdown; a noticeably larger drop than at similar points in earlier years.

**Fig. 4:**
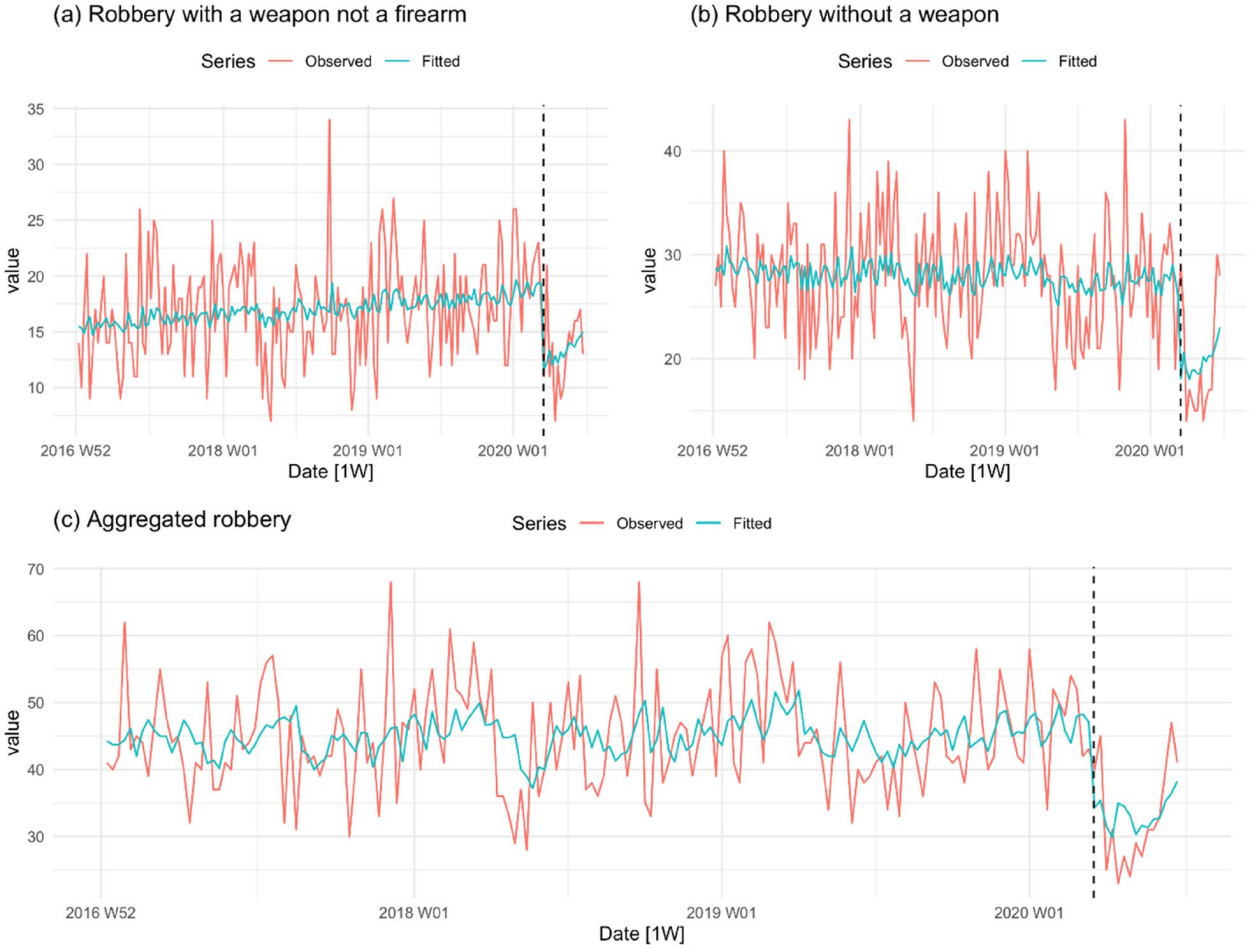
Robbery offences per week in NSW

Figure 4 shows the trends for robbery with a weapon other than a firearm, robbery without a weapon and the two offences combined. During the pre-COVID-19 period, there is a steady upward trend in robbery with a weapon other than a firearm. The lockdown is followed by a marked drop in the level of this offence. Robbery without a weapon appears comparatively stable during the pre-COVID-19 period but shows the same abrupt drop in level after the onset of the COVID-19 lockdown. The combined series strengthens the impression of an abrupt and unexpected fall in robbery.

**Fig. 5:**
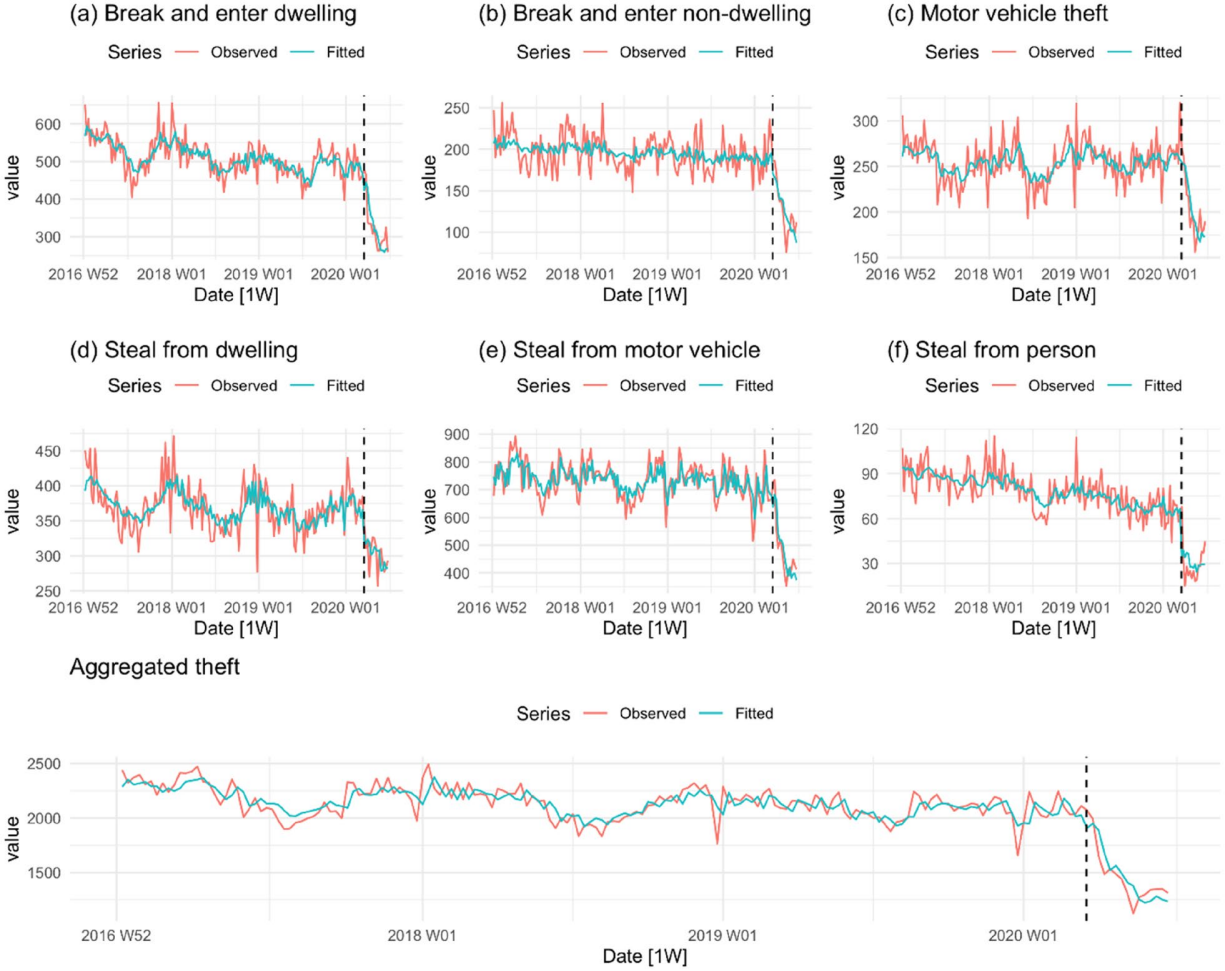
Property offences per week in NSW

Figure 5 shows the trends in theft offences. The pre-lockdown trends for motor vehicle theft and stealing from a dwelling appear stable. However, break and enter dwelling; break and enter non-dwelling; stealing from a motor vehicle; and stealing from the person, all show distinct downward trends in the run-up to the COVID-19 lockdown. Immediately following the lockdown, all series fall. The aggregate series, however, indicates that the response to the lockdown was an accelerated decline in the incidence of theft rather than a sudden fall in the level, as occurred with other offences so far examined.

**Fig. 6:**
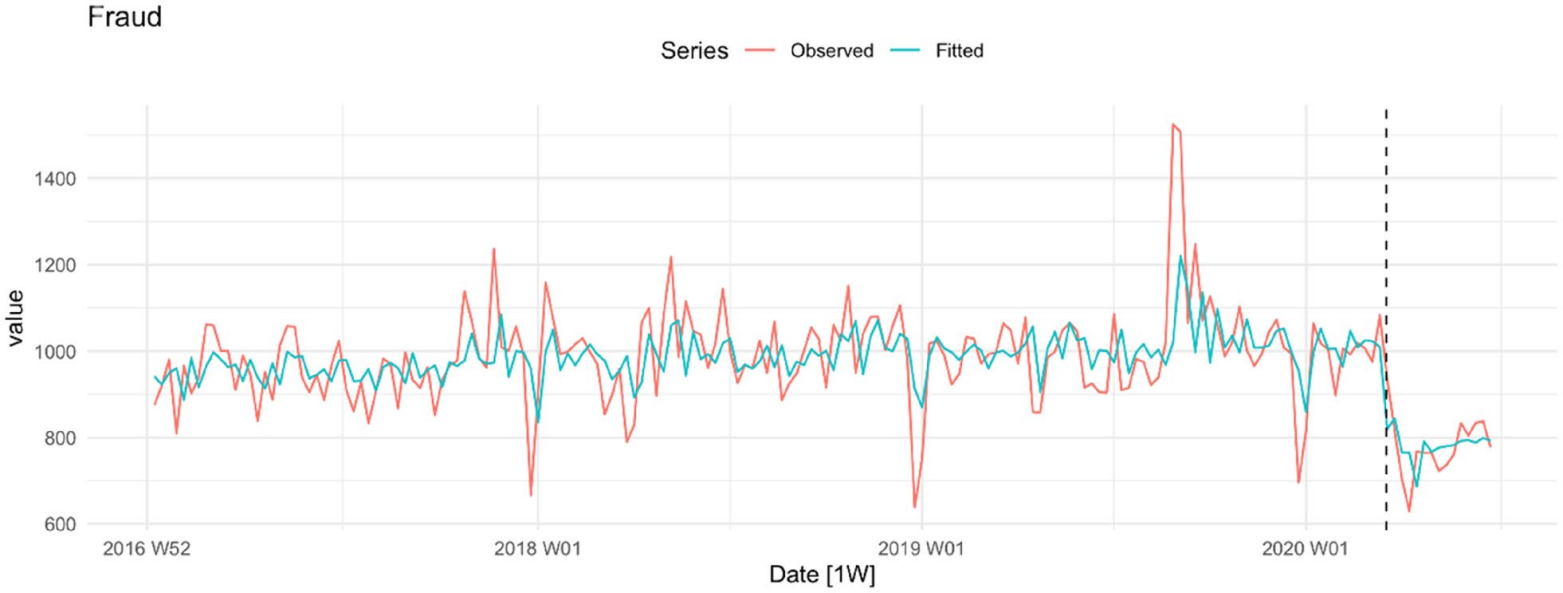
Fraud per week in NSW

Figure 6 shows the trend in fraud. Although there is considerable volatility in the series during the pre-COVID-19 period, there is only faint evidence of an upward trend in the mean level of the series. As with many of the other series we have examined, however, immediately following the lockdown there is a steep drop in the level and very little indication of a return to pre-COVID-19 lockdown levels.

**Fig. 7:**
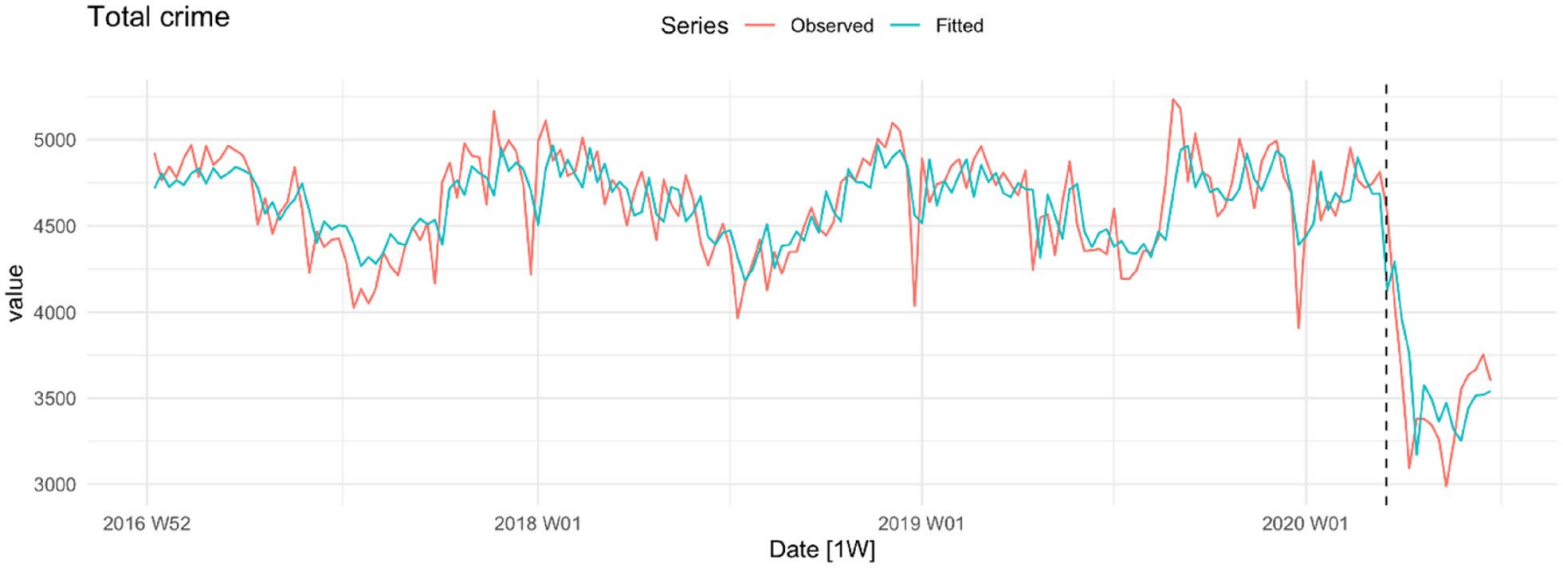
Total crime per week in NSW

Figure 7 shows the observed and fitted trends for all offences combined. In the pre-COVID-19 period, the combined series is running at around 4500 incidents per week. Immediately after the lockdown commenced, the total number of offences fell to below 3500 incidents per week. To determine whether the impressions generated by Figs. 2, 3, 4, 5, 6 and 7 are statistically significant we turn to the results of the statistical analysis, which are shown in Table 2 below.

**Table 2:** Model parameter estimates and final ARIMA specification

Crime type/group	Pre-lockdown trend	Level change	Trend change	Ljung–Box test	ARIMA specification	RMSE	MAE
Domestic assault	0.417 (0.300)	− 38.1 (35.100)	2.88 (4.100)	0.738	(5,0,0) (1,0,0)	42.99	31.60
Non DV assault	0.001 (0.235)	− 154*** (46.200)	4.58 (5.150)	0.144	(1,0,2) (0,0,1)	46.25	36.77
Indecent assault/act of indecency	0.102* (0.044)	− 48.8*** (14.900)	3.44* (1.740)	0.560	(2,0,2) (1,0,1)	21.57	16.76
Sexual assault	0.071 (0.048)	− 36.9** (13.500)	3.47* (1.50)	0.725	(0,0,2) (0,0,1)	17.17	13.75
Aggregated sexual/indecent assault	0.161 (0.099)	− 85.7*** (26.1)	7.56** (2.92)	0.139	(1,0,0) (0,0,1)	32.44	24.65
Robbery with a weapon not a firearm	0.018* (0.008)	− 6.71** (2.59)	0.169 (0.287)	0.410	(0,0,1) (1,0,0)	4.37	3.52
Robbery without a weapon	− 0.009 (0.011)	− 9.6** (3.40)	0.324 (0.381)	0.559	(0,0,1) (1,0,0)	5.50	4.47
Aggregated robbery	0.006 (0.019)	− 14.7** (4.95)	0.498 (0.543)	0.563	(0,0,5) (1,0,0)	7.32	5.79
Break and enter (dwelling)	− 0.505** (0.169)	− 23.4 (30.5)	− 12.7*** (3.37)	0.927	(1,0,1) (1,0,0)	35.33	27.85
Break and enter (non-dwelling)	− 0.111** (0.039)	− 17.5 (12.9)	− 5.38*** (1.50)	0.460	(0,0,1) (1,0,1)	20.00	16.26
Motor vehicle theft	0.003 (0.079)	− 16.1 (17.6)	− 4.81* (2.36)	0.568	(3,0,0) (1,0,1)	20.24	15.21
Steal from motor vehicle	− 0.353* (0.175)	− 35.1 (48.1)	− 22.3*** (5.31)	0.702	(2,0,1)	53.17	42.40
Steal from dwelling	− 0.140 (0.119)	− 43.5* (18.9)	− 1.73 (2.04)	0.613	(2,0,2) (1,0,0)	26.23	19.86
Steal from person	− 0.171*** (0.029)	− 30.6*** (7.49)	− 0.011 (0.784)	0.766	(4,0,0) (1,0,0)	10.18	8.09
Aggregated theft	− 1.270** (0.428)	− 136 (98.2)	− 49.4*** (10.7)	0.065	(2,0,0) (1,0,0)	102.77	79.48
Fraud	0.471* (0.220)	− 255*** (65.9)	1.91 (7.39)	0.872	(0,0,1) (0,0,1)	92.52	64.91
Total crime	− 0.129 (0.819)	− 635** (202)	− 38.8 (22.2)	0.822	(1,0,0) (1,0,0)	203.64	160.62

The offences included in the study are listed in column one. The next two columns show the underlying trends for each offence category in the pre-lockdown period and their associated p-values. The second two columns show the change in the level of each offence after the lockdown and its associated p-value. The third pair of columns provide the same information but in relation to a change in trend rather than a change in level. Column eight provides the results of the Ljung Box test for testing the presence of autocorrelation in the model residuals. None of the Ljung–Box test results is statistically significant, indicating that there are no problems with autocorrelated residuals in any of the models. The next column provides the ARIMA specification. The last two columns provide the root mean square error (RMSE) and mean absolute error (MAE), respectively. In order to provide some context to these goodness of fit measures, we also considered the Interrupted Time Series Analysis (ITSA) model by using the R package its.analysis (English, 2021). The results from the ITSA/ARIMA comparisons in terms of some model accuracy metrics are available in Additional file 1. We would also like to highlight that all the series under the ITSA model are estimated independently to each other but those under the ARIMA specification are subjected to the hierarchical structure constraints.

Inspection of column 2 (pre-lockdown trend) reveals that the upward trends we observed in connection with indecent assault/act of indecency (Fig. 3), robbery with a weapon not a firearm (Fig. 4), and fraud (Fig. 6) during the pre-COVID-19 lockdown are all statistically significant. The pre-lockdown (downward) trends observed in connection with break and enter (dwelling and non-dwelling); stealing from a motor vehicle, stealing from the person, and aggregate theft (Fig. 5) are also statistically significant. The first question of interest, then, is whether the COVID-19 lockdown made any difference to these pre-existing trends. Inspection of columns four and five reveals that there were. We observe significant falls in the mean level of the series in the case of indecent assault/act of indecency (down about 49 offences), robbery with a weapon not a firearm (down 7 offences), steal from the person (down 31 offences) and fraud (down 255 offences).

The second question of interest is whether any of the offences that were stable during the pre-lockdown period also exhibited a drop in level. The answer to this question is also ‘yes’. In the post-lockdown period there were significant declines in non-DV assault (down 154 offences), sexual assault (down 40 offences), aggregated sexual/indecent assault (down 86 offences), robbery without a weapon (down 10 offences), aggregated robbery (down 15 offences), steal from a dwelling (down 44 offences) and total crime (down 635 offences). Columns six and seven provide information about the effect of the COVID-19 lockdown on changes in the trend of each offence. The short follow-up period cautions against putting too much weight on these results but it is worth noting that break and enter dwelling, break and enter non-dwelling, motor vehicle theft, stealing from a motor vehicle and aggregated theft all show a significantly faster rate of decline in the post-COVID-19 lockdown period. In summary, all the coefficients associated with a change in the level of the series in the post-lockdown period are negative, 11 of the 17 tests for a change in level are statistically significant and the falls in certain categories of crime (e.g. non-DV assault, aggregated theft, aggregated sexual/indecent assault) are substantial.

## Discussion and conclusions

The aim of this article is to report the results of a study into the effect of the COVID-19 restrictions on crime in New South Wales (NSW), Australia. Using interrupted time series design as a quasi-experimental approach for evaluating interventions, we were able to control for underlying trend and seasonal pattern with multiple time points. We find no increase in any category of crime, substantial falls in the level of violent sex offences and somewhat more moderate declines in some theft offences (stealing from a dwelling, stealing from the person); and fraud. When all offences are aggregated together, the net effect is a substantial drop in reported crime (approx. 635 fewer offences/week). Although we observed no immediate drop in the mean level of break and enter dwelling; break and enter non-dwelling; motor vehicle theft; stealing from a motor vehicle; or aggregated theft, some of these offences do show signs of declining at a more rapid rate than their pre-COVID-19 trends would have suggested. For most of the crime series, the interrupted time series ARMIA model provided adequate fit. For some categories of crime such as robbery, the ARIMA model did not provide as good a fit visually due to a lack of serial dependence in the data. It may be worthwhile exploring in the future, if methods based on different architectures such as machine learning would give better fit to the data as suggested by one of the reviewers.

The decline in violent crime is interesting because victims of violent crime (especially sexual assault and domestic violence) often delay reporting their experience to police. The same is true of fraud, although in this case the reason for delay typically relates to the time it takes for the victim to discover the offence. The likely explanation for the quick response of violent offences to the COVID-19 lockdown is that victims of violence are generally more willing to report the offence when the offender is a stranger than when the offenders is someone known to them (Australian Bureau of Statistics, 2021). These victims may have been willing to report the offence soon after experiencing it. If this is true, we may yet see further reductions in violent crime, especially sex and domestic violence offences, as those who know their offenders begin reporting to police. It is possible the drop in reports of fraud stems from instances where the offence is quickly discovered (e.g. fraudulent use of credit cards). The closure of retail businesses would have reduced the opportunities for these offences.

Our results are consistent with those found by Payne et. al. (2020, 2021) in Queensland, Australia. They appear more substantial and more consistent than those found in the earlier-reviewed studies in the U.S. They appear less substantial than those observed by Halford et. al. (2020) in the United Kingdom (U.K), although that study focussed on one area rather than the whole of the U.K. The most noteworthy difference between the current findings and those in other countries is that we observed no change in reported cases of domestic assault. One possible explanation for this, canvassed above, is that there may be a lag in the reporting or recording of this offence. The lockdown could have increased the lag in reporting by making it more difficult for victims of domestic violence to leave home and report their victimisation to police. The difficulty with this explanation is that other jurisdictions have seen an increase in domestic assault in response to the restrictions on social movement. Another possibility is that the burdens involved in enforcing compliance with social distancing have delayed recording of domestic assault by police. A third is that compliance with social movement restrictions may have been lower in areas where domestic violence is more prevalent. Regional variations in the effects of the lockdown have been observed by others (McCarthy et al., 2021). Further research is clearly necessary to clarify this unexpected observation.

All non-experimental studies have their limitations when it comes to testing causal hypotheses. We cannot completely rule out alternative causes for the changes in crime that we observed. However, as the only legislative interventions at the time were a series of public health orders and the lockdown, it is highly unlikely that are other causes at the state level having a significant impact on the crime trends. Another methodological limitation is the use of piecewise linear function in the interrupted time series model, which may be restrictive for certain series. More flexible functional forms could be considered, at the cost of losing straightforward interpretability of model estimates. Ultimately it is the convergence of evidence from multiple studies rather than the results of any one study that will give us a clearer picture of the effect of restricting social movement on crime.

At least in the short run, the supply of opportunities and incentives for involvement in crime appears to be much more important in driving crime trends than the supply of motivated offenders. Between March 2020 and June 2020, the number of unemployed persons in Australia rose from 716,000 (5.2%) to 993,700 (7.5%), an increase in relative terms of around 44 per cent (Australian Bureau of Statistics, 2020). Job Seeker payments as a form of social assistance were increased to offset the effect of this growth in unemployment, however, the rapid and substantial increase in unemployment would have provided plenty of motivation to commit income-generating crimes such as burglary and robbery. The absence of any upward trend is a testament to the salience of opportunity as a driver of crime. Businesses with a large cash turnover (e.g., hotels, clubs, shopping centres) were all closed. Pedestrian traffic dramatically declined. Fewer vehicles were parked on the street in locations where they could easily be broken into or stolen. And stay-at-home directions would have left far fewer houses unguarded during the day. The only surprise was the decline in fraud, the electronic forms of which could still be pursued at home. It may be that much of the fraud cases recorded by police occur at the point of sale (e.g., with stolen or counterfeit credit cards).

The effect of restricting social movement on crime carries important implications for policy makers. This is not because restrictions on social movement have emerged as an effective means of controlling crime but because their effect underscores the potential to control crime through restrictions of criminal incentives and opportunities. Criminologists have long known this, but the primary response of Government to instances of lawbreaking all too often remains one of sanctioning the lawbreaker in the expectation that this will discourage further involvement in crime. Harsh penalties, however, have little specific or general deterrent effect (Chalfin & McCrary, 2017; Liedka et al., 2006; Nagin et al., 2009): regulatory reforms that make it harder to sell stolen goods, harder to steal cars, harder to commit fraud and/or which remove the triggers for violent behaviour (e.g. alcohol abuse), on the other hand, have been shown in numerous studies to be an effective form of crime control.

This is not to say larger macrosocial and macroeconomic factors may not also influence crime. Although the effects of COVID-19 on crime are generally positive, it would be a mistake to dismiss the possibility of a rise in crime over the longer term. We have been examining the short-term trends. Crime tends to rise during periods of economic contraction and fall during periods of economic growth (Bushway et al., 2013; Cook & Zarkin, 1985). In some countries the economic effects of the pandemic may last much longer than the lockdown. The next step is to try and gain a deeper understanding of why the COVID-19 lockdown had such variable effects in different countries and locations. To obtain this understanding, we need a site where detailed records have been kept on the spatiotemporal distribution of criminal opportunity before and after the introduction of restrictions on social and business activity.

## Supplementary Information

**Additional file 1: Table S1.** RMSE and MAE for ARIMA and ITSA models.

## Data Availability

The data used in the study are available upon request.
