# Supplementary material for: The impact of the COVID-19, social distancing, and movement restrictions on crime in NSW, Australia
Source: Crime Sci. 2021 Oct 24;10(1):24. doi: 10.1186/s40163-021-00160-x (PMC8542413; doi:10.1186/s40163-021-00160-x)
Supplement: Supplementary file 1 — Additional file 1: Table S1. RMSE and MAE for ARIMA and ITSA models. [file 40163_2021_160_MOESM1_ESM.docx]

# Additional file 1: The impact of COVID-19 on crime

Table S1: RMSE and MAE for ARIMA and ITSA models

| Crime type | ARIMA_RMSE | ITSA_RMSE | ARIMA_MAE | ITSA_MAE |
| --- | --- | --- | --- | --- |
| Domestic assault | 42.99 | 53.55 | 31.60 | 41.62 |
| Non domestic assault | 46.25 | 53.09 | 36.77 | 42.01 |
| Fraud | 92.52 | 96.68 | 64.91 | 66.04 |
| Robbery with a weapon not a firearm | 4.37 | 4.46 | 3.52 | 3.60 |
| Robbery without a weapon | 5.50 | 5.54 | 4.47 | 4.51 |
| Aggregated robbery | 7.32 | 7.62 | 5.79 | 6.07 |
| Indecent assault/act of indecency | 21.57 | 23.58 | 16.76 | 17.82 |
| Sexual assault | 17.17 | 17.99 | 13.75 | 14.17 |
| Aggregated sexual assault | 32.44 | 35.61 | 24.65 | 29.75 |
| Break and enter (dwelling) | 35.33 | 38.34 | 27.85 | 29.75 |
| Break and enter (non-dwelling) | 20.00 | 20.44 | 16.26 | 16.39 |
| Motor vehicle theft | 20.24 | 21.38 | 15.21 | 16.59 |
| Steal from dwelling | 26.23 | 29.27 | 19.86 | 22.06 |
| Steal from motor vehicle | 53.17 | 54.63 | 42.40 | 42.71 |
| Steal from person | 10.18 | 11.59 | 8.09 | 9.12 |
| Aggregated theft | 102.77 | 107.77 | 79.48 | 79.20 |
| Aggregated crime | 203.64 | 212.78 | 160.62 | 160.92 |
